# NIR-switchable local hydrogen generation by tandem bimetallic MOFs nanocomposites for enhanced chemodynamic therapy

**DOI:** 10.1093/rb/rbad097

**Published:** 2023-10-31

**Authors:** Jun Zhong, Xiang Zheng, Yuan Wen, Yuewei Li, Jianting Zhang, Ranjith Kumar Kankala, Shibin Wang, Aizheng Chen

**Affiliations:** Institute of Biomaterials and Tissue Engineering, Huaqiao University, Xiamen 361021, China; Fujian Provincial Key Laboratory of Biochemical Technology, Huaqiao University, Xiamen 361021, China; Institute of Biomaterials and Tissue Engineering, Huaqiao University, Xiamen 361021, China; Fujian Provincial Key Laboratory of Biochemical Technology, Huaqiao University, Xiamen 361021, China; Institute of Biomaterials and Tissue Engineering, Huaqiao University, Xiamen 361021, China; Fujian Provincial Key Laboratory of Biochemical Technology, Huaqiao University, Xiamen 361021, China; Institute of Biomaterials and Tissue Engineering, Huaqiao University, Xiamen 361021, China; Fujian Provincial Key Laboratory of Biochemical Technology, Huaqiao University, Xiamen 361021, China; Institute of Biomaterials and Tissue Engineering, Huaqiao University, Xiamen 361021, China; Fujian Provincial Key Laboratory of Biochemical Technology, Huaqiao University, Xiamen 361021, China; Institute of Biomaterials and Tissue Engineering, Huaqiao University, Xiamen 361021, China; Fujian Provincial Key Laboratory of Biochemical Technology, Huaqiao University, Xiamen 361021, China; Institute of Biomaterials and Tissue Engineering, Huaqiao University, Xiamen 361021, China; Fujian Provincial Key Laboratory of Biochemical Technology, Huaqiao University, Xiamen 361021, China; Institute of Biomaterials and Tissue Engineering, Huaqiao University, Xiamen 361021, China; Fujian Provincial Key Laboratory of Biochemical Technology, Huaqiao University, Xiamen 361021, China; Fujian Provincial Key Laboratory of Biomass Low-Carbon Conversion, Huaqiao University, Xiamen 361021, China

**Keywords:** chemodynamic therapy, nanozyme, bimetallic MOFs, gas therapy

## Abstract

The inadequate quantity of hydrogen peroxide (H_2_O_2_) in cancer cells promptly results in the constrained success of chemodynamic therapy (CDT). Significant efforts made throughout the years; nevertheless, researchers are still facing the great challenge of designing a CDT agent and securing H_2_O_2_ supply within the tumor cell. In this study, taking advantage of H_2_O_2_ level maintenance mechanism in cancer cells, a nanozyme-based bimetallic metal-organic frameworks (MOFs) tandem reactor is fabricated to elevate intracellular H_2_O_2_ levels, thereby enhancing CDT. In addition, under near-infrared excitation, the upconversion nanoparticles (UCNPs) loaded into the MOFs can perform photocatalysis and generate hydrogen, which increases cellular susceptibility to radicals induced from H_2_O_2_, inhibits cancer cell energy, causes DNA damages and induces tumor cell apoptosis, thus improving CDT therapeutic efficacy synergistically. The proposed nanozyme-based bimetallic MOFs-mediated CDT and UCNPs-mediated hydrogen therapy act as combined therapy with high efficacy and low toxicity.

## Graphical Abstract

**Figure:**
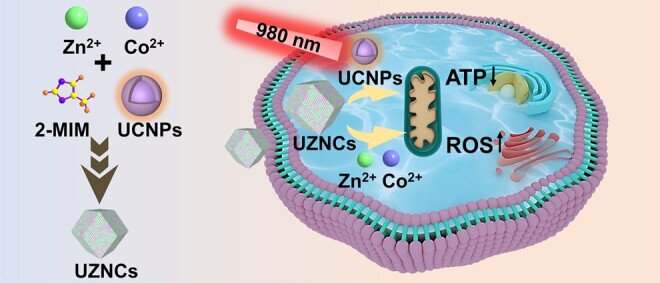

## Introduction

Lately, chemodynamic therapy (CDT) has garnered considerable interest because of its triggering rooted in internal triggers within cancerous cells [1–4]. CDT could utilize the Fenton reaction mediated by Fenton ions (Fe^2+^, Cu^2+^ or Mn^2+^), converting high concentrations of hydrogen peroxide (H_2_O_2_) with lower oxidative activity in tumor cells into hydroxyl radicals (^•^OH) that cause serious oxidative damage to cells, inducing apoptosis and necrosis [5–8]. Due to the vigorous metabolism in tumor cells, during the metabolic process, compared with normal cells, the reduction of oxygen concentration is lower, the pH is decreased and H_2_O_2_ is overexpressed [9–11]. Yet, in the course of cancer therapy, the optimal CDT outcome is rarely realized, primarily as the level of H_2_O_2_ in cancerous cells proves insufficient to engender sufficient ^•^OH for oxidative harm and programmed cell death [12–14]. Consequently, novel approaches are imperative to enhance the H_2_O_2_ concentration in tumor cells to render CDT efficiently.

Within cancer cells, the H_2_O_2_ level maintains a dynamic equilibrium, managed by diverse antioxidant enzymes and biomolecules [superoxide dismutase (SOD), glutathione (GSH)]. Notably, superoxide anion ($O_{2}^{•-}$) is enzymatically converted by SOD to produce H_2_O_2_ [15–17]. The ensuing H_2_O_2_ can undergo transformation into a remarkably deadly ^•^OH through a reaction reminiscent of Fenton, culminating in a significantly amplified CDT. However, natural enzymes usually suffer from poor stability and high cost, which limits further application. Luckily, artificial nanozymes, with the well-developed synthesis strategies and clearly understood mechanisms, can perfectly address above-mentioned concerns [18–23]. Specifically, metal-organic frameworks (MOFs)-derived nanozymes have lately gained substantial focus owing to their porous nanoarchitecture and adaptability [24–26]. Notably, Zn^2+^ could increase mitochondrial $O_{2}^{•-}$ by the inhibiting mitochondrial electron transport chain [27]; hence, if Zn^2+^ and the Fenton process combine as a consecutive reactor, in theory, it will considerably heighten the amounts of $O_{2}^{•-}$ and H_2_O_2_ within cancer cells, consequently boosting the CDT impact. Therefore, if Zn^2+^ and Fenton ions can be integrated into the MOFs, the current shortage of H_2_O_2_ can be largely relieved, thereby exerting an enhanced CDT efficacy.

Recently, hydrogen (H_2_) has received attention for its anti-cancer potential [28, 29]. Typical administration includes H_2_ inhalation and oral H_2_-rich water, but all suffer from poor efficacy since a minimum amount of H_2_ could be carried to lesion [30, 31]. Some nanocarriers with strong affinity for H_2_ are also used to bring more H_2_ to tumor sites [28, 32, 33]. Nevertheless, these nanocarriers usually have low loadings and have the risk of early leakage of H_2_. Therefore, developing a nanomaterial capable of *in situ* sustainable release of H_2_ is inevitable to move H_2_ therapy one step further. At present, photocatalytic MOFs with H_2_ production attributes have undergone rapid advancement because of their modifiable framework, extensive surface zone, elevated porosity, etc. [34–36]. However, short wavelength light is required to produce H_2_ [37, 38], which suffers from poor tissue penetration, therefore greatly limiting their biomedical uses. Since lanthanide-doped upconversion nanoparticles (UCNPs) can be stimulated via near-infrared (NIR) irradiation and give off short wavelength emissions down to ultraviolet (UV), UCNPs can be used as nanotransducer and coupled with photocatalytic MOFs to produce H_2_ under NIR excitation [39–41]. To this end, we devoted to design and construct a new nanozyme based on UCNPs.

**Scheme 1.**
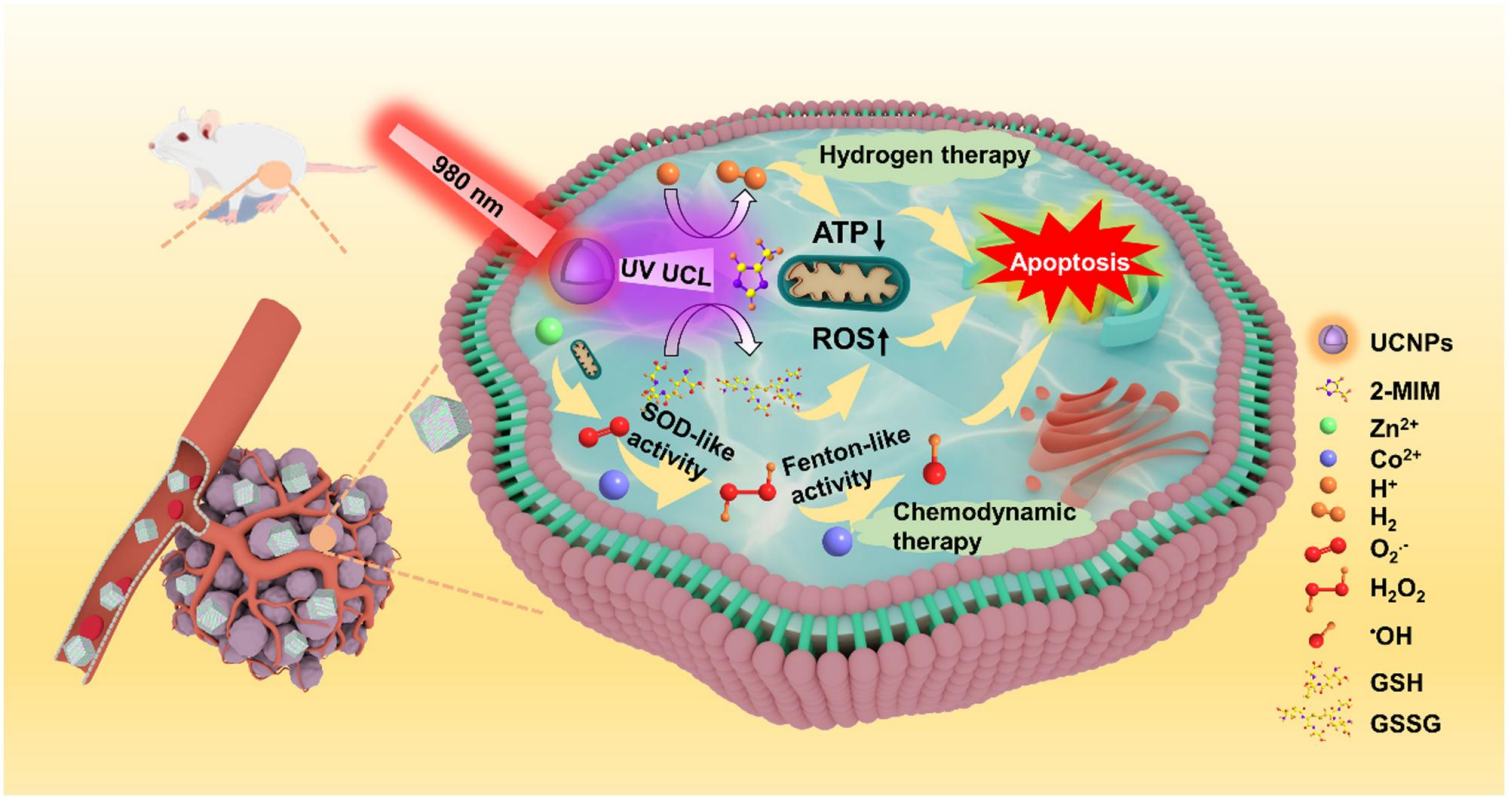
Schematic illustration of Zn–Co ZIF bimetallic MOFs nanoparticles tandem reactor-induced enhanced chemodynamic therapy and UCNPs-mediated hydrogen therapy by NIR-to-Ultraviolet (UV) conversion.

To this end, we designed and constructed the H_2_-producing nanozyme by incorporating UCNPs and Zn–Co ZIF together, forming UCNPs@Zn–Co ZIF nanocomposites (UZNCs). After UZNCs enter the lysosome, under the excitation of NIR light, H_2_ is first produced to accelerate lysosomes’ escape. Subsequently, the generated H_2_ inhibited mitochondrial electron transport, thereby inhibiting the synthesis of intracellular adenosine triphosphate (ATP), making the cell more susceptible to CDT. At the same time, under a tumor acidic environment, the nanozyme Zn–Co ZIF disintegrated to release Zn^2+^ and Co^2+^, simultaneously, Zn^2+^ triggers mitochondria to generate increased $O_{2}^{•-}$, which is swiftly altered into H_2_O_2_ by Co^2+^, increasing the level of H_2_O_2_ in tumor cells. Thus, with abundant H_2_O_2_ and higher susceptibility of tumor cells, enhanced therapeutic efficacy of CDT could be expected (Scheme 1, Supplementary Fig. S1).

## Materials and methods

### Materials

Zinc nitrate hexahydrate (Zn(NO_3_)_2_·6H_2_O), reduced GSH, 2-methylimidazole (2-MIM) and cobalt nitrate hexahydrate (Co(NO_3_)_2_·6H_2_O) were acquired from Sinopharm Chemical Reagent Co. (Shanghai, China). Octadecenylamine (OA) was sourced from Aladdin Reagents Co. (Shanghai, China). 1,3-Diphenylisobenzofuran, nitrotetrazolium blue chloride (NBT) and fluorescein isothiocyanate (FITC) isomer were obtained from Admas-beta Reagents Co. (Shanghai, China).

### Measurements and characterizations

Transmission electron microscopy (TEM) analysis was conducted using an FEI/Talos F200X G2 instrument from Thermo Scientific (Waltham, USA). Scanning electron microscopy (SEM) was carried out using a Hitachi SU5000 instrument. X-ray diffraction (XRD) analysis was utilizing a Bruker D8 Advance XRD instrument with Cu Kα radiation, manufactured by Bruker AXS in Germany. To determine the absorption properties of the nanomaterials, ultraviolet-visible (UV–Vis) spectrophotometry was employed, utilizing a TU-1810 instrument manufactured by Purkinje in China. Confocal laser scanning microscopy (CLSM) images were captured under a Leica TCS SP8 confocal microscope from Germany.

### Synthesis of Zn–Co ZIF

Zn–Co ZIF nanoparticles were prepared in adherence to the procedure outlined in the referenced literature [42]. Initially, 0.1116 g of cobalt nitrate hexahydrate and 0.1674 g of zinc nitrate hexahydrate were thoroughly dissolved in methanol (4.5 ml). Subsequently, this prepared solution was carefully combined with a 4.5 ml methanol solution containing 0.1848 g of 2-MIM, followed by a 10-min sonication period. The resulting purple mixture was then subjected to magnetic stirring for a duration of 4 h. Afterward, the resulting pellet was separated through centrifugation, subjected to three successive methanol washes and subsequently placed under vacuum conditions at 60°C overnight.

### Synthesis of OA-UCNPs

The core–shell UCNPs synthesis was adjusted from the earlier protocol [43]. In a conventional approach, a blend containing 0.01 mmol TmCl_3_·6H_2_O, 0.4 mmol YbCl_3_·6H_2_O, 1.59 mmol YCl_3_·6H_2_O, 12 ml of OA and 1-octadecene was prepared. This mixture was carefully homogenized, and in the absence of oxygen, it was heated to 120°C for a duration of 1 h. Afterward, it was cooled down to ambient temperature. To this solution, a rapid addition of NaOH and NH_4_F was performed. The mixture's temperature was promptly elevated to 120°C for a duration of 30 min to guarantee thorough elimination of methanol. Ultimately, the solution was subjected to heating at 300°C within an Ar environment for 1 h. The resultant UCNPs underwent purification via a series of centrifugation stages and were subsequently suspended in 20 ml of cyclohexane. The core–shell UCNPs were synthesized via secondary pyrolysis, building upon the inner core UCNPs. For this, 0.5 mmol of YCl_3_·6H_2_O, 15 ml of 1-octadecene and 6 ml of OA were introduced into a flask and blended meticulously. The concoction was reacted at 120°C for 1 h until it fully dissolved. Upon achieving a homogeneous solution, the heating was discontinued, and then, at ambient temperature, 10 ml of the cyclohexane solution comprising UCNPs was introduced. Following the evaporation of cyclohexane, a mixed solution containing NH_4_F, NaOH and methanol was slowly introduced. The temperature was then gradually increased, allowing the gradual evaporation of methanol. Following that, it underwent degassing at 120°C for 30 min and was subsequently heated while in an argon atmosphere, holding it at 300°C for 1 h. Subsequently, the heating process was concluded. The core–shell UCNPs were obtained by precipitating them with ethanol, collecting them via centrifugation and ultimately dispersing them in 10 ml of cyclohexane for subsequent utilization.

### Ligand exchange of PVP with OA-UCNPs

Since oleic acid ligands on UCNPs surface are not conducive to MOFs growth, PVP was used to replace oleic acid ligands to create a suitable surface for MOFs growth [44–46]. The OA-UCNPs obtained above were ethanol-precipitated and centrifuged to remove surface oleic acid. Subsequently, they were re-suspended in 25 ml of chloroform. Following that, 1 g of PVP was included in the chloroform solution of UCNPs, dispersed through ultrasonic means to generate a uniform solution and then magnetically stirred overnight. Ultimately, the surplus PVP was eliminated through five rounds of centrifugation with ethanol, and the resulting precipitate was re-suspended in ethanol for subsequent application.

### Synthesis of UCNPs@Zn–Co ZIF

To initiate the process, 0.01 g of the previously synthesized PVP-UCNPs was dispersed in a mixed solution comprising 0.1116 g of cobalt nitrate hexahydrate and 0.1674 g of zinc nitrate hexahydrate, with a subsequent 30-min magnetic stirring period to facilitate the enrichment of metal ions on the surface of the PVP-UCNPs. After this, the solution, comprising 0.1848 g of 2-MIM and 4.5 ml of methanol, was added to the previously mentioned mixture. After 4 h of continuous stirring of the mixed solution, a purple precipitate was formed and subsequently separated via centrifugation. The resulting precipitate was then subjected to three successive methanol washes and dried under vacuum conditions at 60°C overnight.

### Measurement of intracellular ATP and reactive oxygen species concentrations

Intracellular ATP and reactive oxygen species (ROS) levels were assessed using specific detection kits. To begin, 4T1 cells were irradiated for 10 min. After a 3-h interval, 4T1 cells underwent lysis, and the resultant supernatant was obtained by centrifugation. Subsequently, the quantities of intracellular ATP and ROS were assessed using the relevant assay kits.

### Assessment of *in vitro* hydrogen generation

Quantitative monitoring of hydrogen concentration was achieved via gas chromatography. In a simulated solution, a reaction mixture containing nanoparticles and GSH was stirred in nitrogen atmosphere. The manageability of NIR-induced hydrogen generation was evaluated by cyclically activating and deactivating the laser.

4T1 cells were cultured with nanoparticles for 8 h and then subjected to irradiation. Then, 4T1 cells were lysed through two quick freeze-thaw cycles. The ensuing supernatant was gathered via centrifugation, and the hydrogen concentration was gauged using gas chromatography.

### *In vitro* assessment of cell viability

Specifically, 4T1 cells were cultivated under appropriate conditions for 24 h. Fresh DMEM containing various nanomaterials (UCNPs, Zn–Co ZIF and UCNPs@Zn–Co ZIF) was introduced to each well. After 24 h of incubation, remove the DMEM solution with nanomaterials. Then, 100 μl of serum-free medium, which included a CCK-8 solution (10% CCK-8), was introduced into each well.

### Staining for live and dead cells

Identification of live and deceased cells was achieved through calcein/PI dual staining and visualization was carried out using CLSM. In a typical procedure, 4T1 cells were placed and cultivated under appropriate environments. 4T1 cells were further cultured with various nanomaterials (UCNPs, Zn–Co ZIF and UCNPs@Zn–Co ZIF) for 24 h. After the removal of the medium and three washes with phosphate buffered saline (PBS), they were stained using calcein and propidium iodide (PI).

### Flow cytometry assessment of apoptosis

Initially, 4T1 cells were placed and incubated under suitable conditions. They were subsequently cultured with various nanomaterials (UCNPs, Zn–Co ZIF and UCNPs@Zn–Co ZIF). Subsequently, the cells were stained with Annexin V-FITC Kit.

### Animal model

Animal experiments were performed following the guidelines sanctioned by the Experimental Animal Ethics Committee of Huaqiao University (No. A2020029). Female BALB/C mice, aged 5–8 weeks and weighing around 20 g, were procured from Shanghai Laboratory Animal Co., Ltd. These mice were kept in standard conditions. Then, 4T1 cells were injected into the mice.

### *In vivo* biodistribution

The *in vivo* biodistribution of nanoparticles was evaluated. UCNPs, Zn–Co ZIF and UCNPs@Zn–Co ZIF in saline were injected via the tail vein. Mice were sacrificed after 4 h, 12 h, 24 h and 14 days post-injection, and their organs and tumors were harvested for analysis, which were carried out after the tissue had been thawed with chloronitrate acid, allowing for the evaluation of the biodistribution of UCNPs, Zn–Co ZIF and UCNPs@Zn–Co ZIF.

### *In vivo* biocompatibility assay

The *in vivo* biocompatibility of nanoparticles was assessed using female Kunming mice (average body weight of 20 g). UCNPs, Zn–Co ZIF and UCNPs@Zn–Co ZIF were suspended in saline and then administered via the tail vein. After a period of 14 days, the mice were euthanized, and their organs were gathered and prepared for hematoxylin and eosin (H&E) staining, which served for subsequent analysis.

### *In vivo* tumor therapy investigations

To evaluate the anti-cancer effects of UCNPs, Zn-Co ZIF and UCNPs@Zn -Co ZIF *in vivo,* saline alone, UCNPs, Zn–Co ZIF and UCNPs@Zn–Co ZIF were injected via tail vein. After this treatment period, tumors and other organs were isolated for further analysis.

### Detection of hydrogen production *in vivo*

*In vivo*, after 4 h of reaction, the mice were irradiated for 10 min, and subsequently, the tumor was removed and swiftly dissolved using grinding. The resulting supernatant was obtained via centrifugation, and the *in vivo* H_2_ production was measured using gas chromatography.

### *In vivo* $O_{2}^{•-}$ staining

Mice were intravenously administered UCNPs, Zn–Co ZIF and UCNPs@Zn–Co ZIF for 12 h. All mice were injected intraperitoneally with dihydroethidium (DHE) (3 mg ml^−1^). The tumors were surgically excised, sliced and, subsequently subjected to staining with DAPI and DHE. Tumor $O_{2}^{•-}$ staining images were obtained under a microscope.

### Histological examination

Following a 14-day treatment period, the mice were euthanized, and tumors and organs were excised for additional analysis. H&E staining was conducted on major organs.

## Results and discussion

### Preparation and characterization of UZNCs

**Figure 1.**
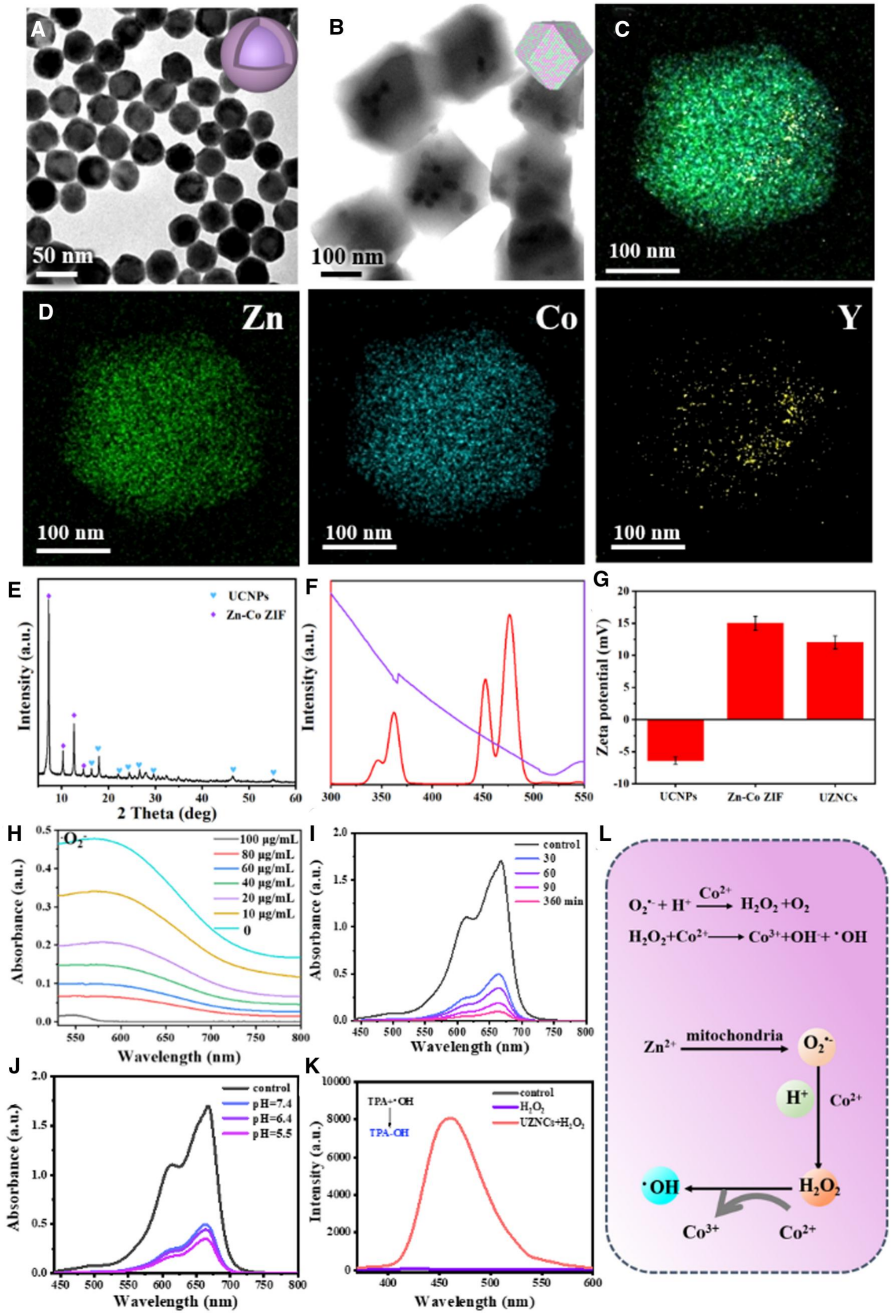
Controlled synthesis and characterization of UZNCs. (**A**) TEM image of UCNPs. (**B**) TEM image of UZNCs. (**C** and **D**) High-angle annular dark-field scanning TEM (HAADF-STEM) elemental mapping images of UZNCs. (**E**) XRD pattern of UZNCs. (**F**) The fluorescence emission spectrum of UCNPs and the UV–Vis absorption spectrum of 2-MIM. (**G**) ζ potentials of UCNPs, Zn–Co ZIF and UZNCs. (**H**) The SOD-mimicking activity of UZNCs. (**I**) The degradation progression of MB at various time intervals. (**J**) The rate of MB degradation under varying pH conditions. (**K**) TPA fluorescence before and after its interaction with UZNCs. (**I**) Schematic diagram of generating $O_{2}^{•-}$ and ^•^OH.

Core–shell UCNPs were prepared using high-temperature coprecipitation methods [43]. NaYF_4_:Yb^3+^/Tm^3+^ UCNP cores were first synthesized, and subsequently, an inert NaYF_4_ shell layer was epitaxially grown. The obtained UCNPs exhibited uniform size and morphology (Fig. 1A). Subsequently, Zn–Co ZIF was synthesized using a straightforward one-step reaction method, resulting in particles with an approximate size of 200 nm (Supplementary Figs S2 and S3). After encapsulating core–shell UCNPs nanoparticles into Zn–Co bimetallic ZIF nanoparticles, dodecahedral UZNCs were obtained. The SEM image revealed that the UZNCs exhibited a consistent morphology with an average particle size of around 200 nm (Supplementary Fig. S4). The ratio of Zn and Co elements in the Zn–Co MOF was 2:1 (Supplementary Fig. S5). From the TEM images, it was observed that UCNPs were distributed inside the Zn–Co ZIF nanoparticles (Fig. 1B); furthermore, Zn and Co elements were uniformly distributed in the outer shell (Fig. 1C and D). X-ray powder crystallography indicated that the obtained nanoparticles were in the composite state composed of UCNPs and ZIF (Fig. 1E). In addition, the hydrodynamic sizes of Zn–Co ZIFs and UZNCs were recorded by dynamic light scattering analysis, and their sizes corresponded to those determined by TEM and SEM (Supplementary Fig. S6). Compared with Zn–Co ZIF, the nitrogen adsorption–desorption curve of UZNCs showed a slight decrease in specific surface area; this reduction was mostly attributed to the presence of UCNP cores, which blocked the internal pores of Zn–Co ZIF (Supplementary Fig. S7). From the ultraviolet-visible (UV–Vis) spectroscopy (Fig. 1F), it is obvious that the absorption of the MOFs shell is fully overlapped with UCNPs emission profile under 980-nm excitation, ensuring the efficient energy transfer and photoreduction properties. The zeta potential switching (from −6.3 to 12.1 mV) before and after Zn–Co ZIF encapsulation further indicated successful bonding (Fig. 1G).

The successful manufacture of UZNCs encourages us to study its various functions. We next investigated how Zn^2+^ and Co^2+^ in bimetallic MOFs react with intracellular substances to generate free radicals. First, the SOD-mimetic property of UZNCs was investigated by the classical nitrotetrazolium blue chloride (NBT) chromogenic approach. Without UZNCs, under UV irradiation, riboflavin and l-methionine generate $O_{2}^{•-}$, reducing NBT to blue methazine. Once nanoparticles were added, the absorbance intensity dropped significantly, indicating the SOD-mimetic activity of UZNCs. Meanwhile, the disproportionation of $O_{2}^{•-}$ by UZNCs was further intensified as the nanoparticle concentration increased, indicating the concentration-dependent catalytic activity of UZNCs (Fig. 1H). Subsequently, the ^•^OH-generating behaviors of UZNCs, critical for CDT, was investigated. Co^2+^ reacts with H_2_O_2_ to produce ^•^OH, which lead to the degradation of methylene blue (MB) and reaction with terephthalic acid (TPA), producing fluorescent 2-hydroxyterephthalic acid. Fast degradation of MB could be observed when H_2_O_2_ was added to UZNCs suspension. The temporal degradation behavior of MB is shown in Fig. 1I. After 360 min, MB was utterly degraded. We also measured the reaction rates of MB degradation and TPA hydroxylation at different pH values. The results showed a little difference in the reaction rate under different pH values (Fig. 1J). Similar results could be observed in the TPA fluorescence experiment (Fig. 1K), further proving the superior Fenton-like catalytic effect of UZNCs. Therefore, we summarized the pathway of UZNCs generating $O_{2}^{•-}$ and ^•^OH, as shown in Fig. 1l.

### *In vitro* anti-cancer research

**Figure 2.**
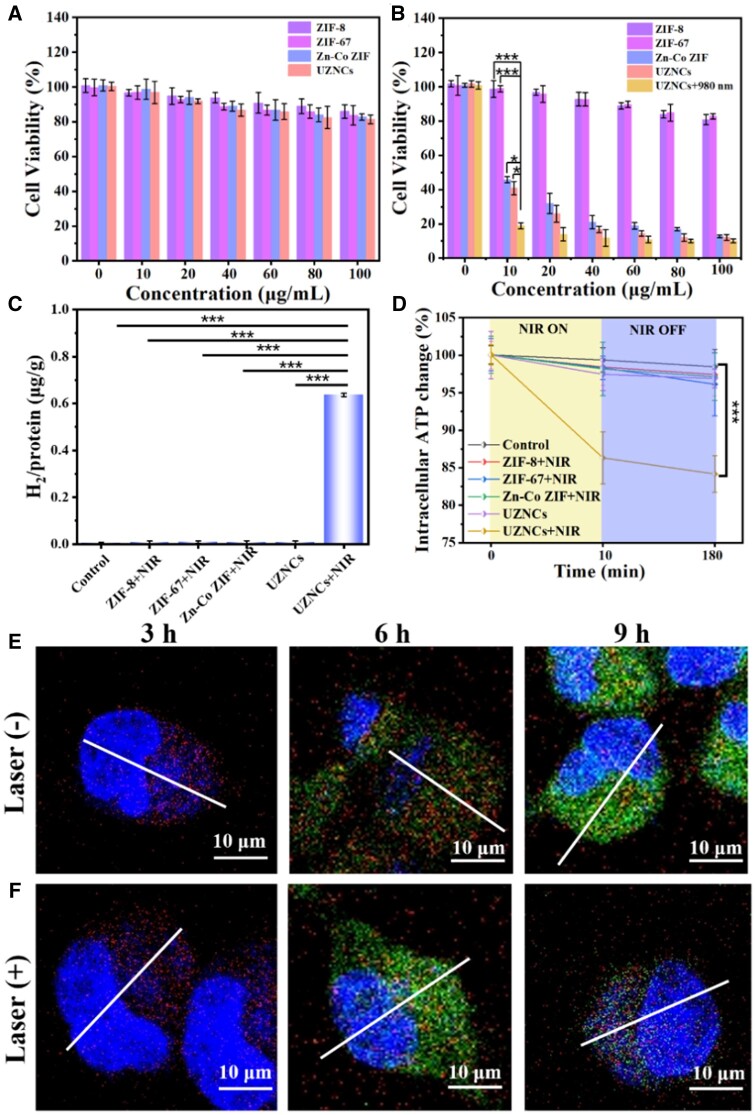
*In vitro* anti-cancer effect, hydrogen production, reduction of adenosine triphosphate (ATP) content and lysosomal escape of UZNCs. Cell viability of (**A**) MCF-10A cells and (**B**) 4T1 cells under different treatments. *P<0.05 and ***P<0.001. (**C**) The intracellular production of hydrogen. ***P<0.001. (**D**) The intracellular production of ATP. ***P<0.001. Fluorescence co-localization pattern (**E**) with laser and (**F**) without laser illumination at different times.

Next, the *in vitro* anti-cancer effects, H_2_ production properties and lysosomal escape properties of UZNCs were explored. The CCK-8 cell counting kit was employed to assess the cell viability of both normal and tumor cell lines subjected to various nanoparticles. The in *vitro* biocompatibility of ZIF-67, ZIF-8, Zn–Co ZIF and UZNCs was evaluated with MCF-10A cells. After co-incubating with these nanoparticles for 24 h, >90% of normal cells survived, indicating that these nanoparticles showed lower cytotoxicity (Fig. 2A). ZIF-67 and ZIF-8 nanoparticles had no noticeable killing effect on 4T1 cells, demonstrating its limited anti-cancer activity. Interestingly, Zn–Co ZIF nanoparticles exhibited potent cytotoxicity against 4T1, killing 80% of 4T1 cells at low concentrations (40 μg ml^−1^) (Fig. 2D). The reason could be that Zn^2+^ and Co^2+^ in Zn–Co ZIF were prone to acid-responsive release in the tumor cells and *in situ* assembled into a tandem reactor, significantly increased $O_{2}^{•-}$ and H_2_O_2_ in tumor cells. Finally, under the biocatalysis of Co^2+^, it is converted into ^•^OH with serious oxidative damage to cells through a Fenton-like reaction, thereby causing tumor cell apoptosis. More importantly, the cell-killing efficiency of UZNCs was further elevated after irradiated with a 980-nm laser, indicating that UZNCs produced a large amount of H_2_ in 4T1 cells after irradiated with a 980-nm laser. In addition, we also investigated the NIR photocatalytic H_2_ production ability of UZNCs. The H_2_ production process of UZNCs was monitored in real time by gas chromatography. In a 100-μM GSH solution, UZNCs could utilize GSH in solution as a reducing agent and exhibit good H_2_ production ability, indicating that they have the potential to produce H_2_*in vivo* for H_2_ therapy (Supplementary Fig. S8). Therefore, we further examined the ability of UZNCs to produce H_2_ intracellularly. Under the irradiation of a 980-nm laser, UZNCs could generate H_2_; however, no H_2_ generation was detected for the control, ZIF-8 + NIR, ZIF-67 + NIR, Zn–Co ZIF + NIR and UZNCs groups, proving that only under NIR irradiation, could Zn–Co ZIFs utilize UV light converted from UCNPs to generate H_2_ (Fig. 2C).

H_2_ inhibits adenosine triphosphate (ATP) synthesis by affecting mitochondrial energy metabolism, thereby causing cancer cells apoptosis for its insufficient energy supply. Therefore, we investigated the effects of UZNCs on intracellular ATP levels with and without NIR irradiation to reveal the mechanism of H_2_ therapy. In addition, the intracellular ATP monitoring results showed that the intracellular ATP level decreased rapidly after being treated with UZNCs, indicating that the tandem reactor was capable of decreasing the intracellular ATP level obviously (Fig. 2D).

The timely escape of lysosomes is essential for maintaining the pharmacological activity of endocytic nanomedicines. We investigated the lysosomal escape behavior of FITC-labeled UZNCs by fluorescent co-locating labeled nanocarriers and lysosomes in 4T1 cells by CLSM imaging. After incubation with UZNCs for 3 h, no green fluorescence was shown in CLSM image with or without NIR, indicating that UZNCs have not been captured by 4T1 cells. After co-incubating for 6 h, it was observed from the CLSM image and the fluorescence intensity map that the fluorescence of FITC superimposed with the red fluorescence from the lysosome, indicating that the UZNCs have successfully entered the lysosome. Interestingly, after co-incubating for 9 h, the fluorescence of FITC in the non-NIR group and the red fluorescence of lysosomes were still in a superimposed state (Fig. 2E and F), indicating that the nanocarriers were still trapped in the lysosome; while the fluorescence of FITC the red fluorescence of lysosomes in the NIR-added group were largely separated, indicating that the nanocarriers have successfully escaped from the lysosome (Supplementary Fig. S9). These results show that H_2_ produced by UZNCs can destroy the lysosome structure under laser exposure.

**Figure 3.**
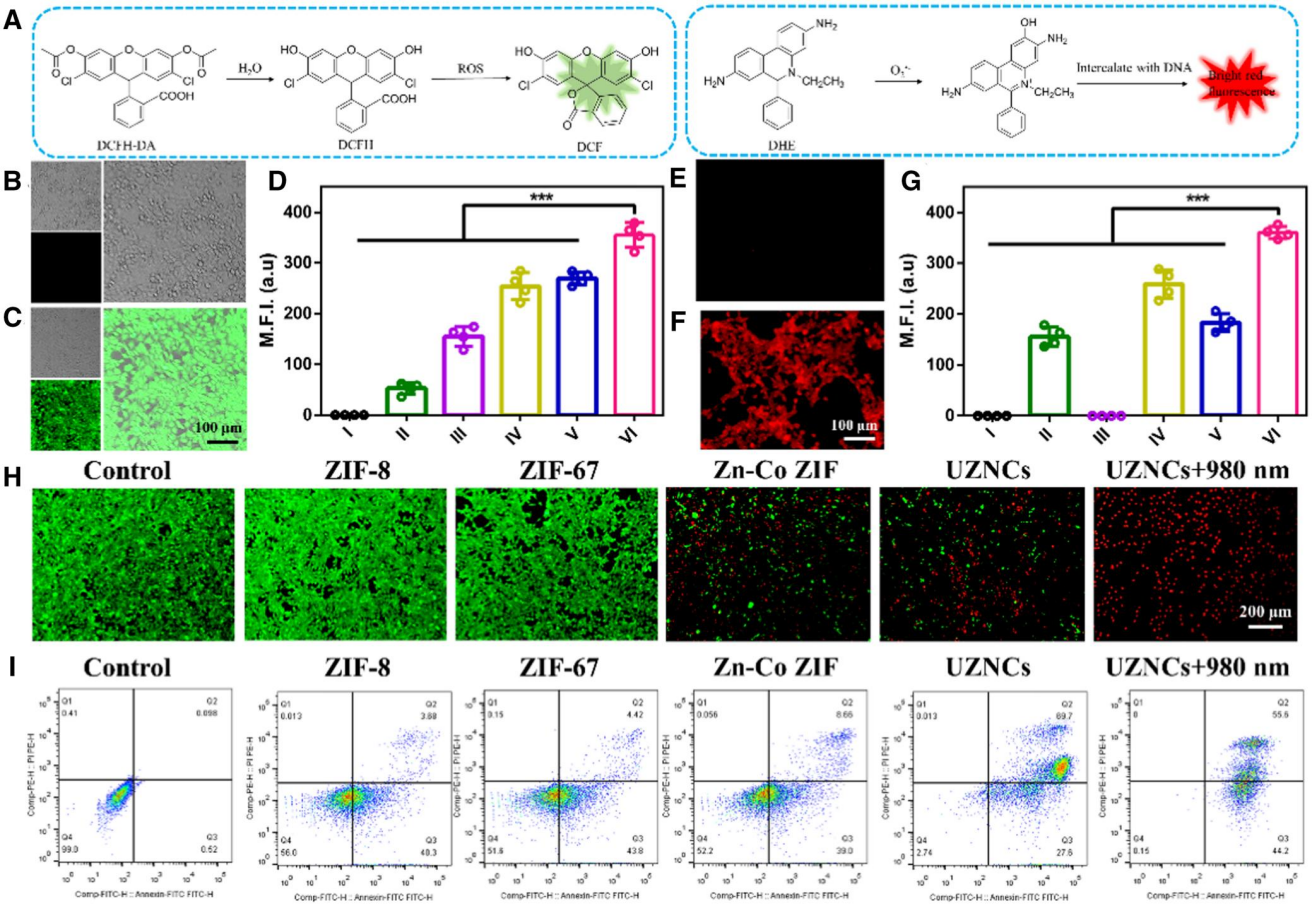
Cellular ROS and $O_{2}^{•-}$ generation in 4T1 cells and toxicity tests. (**A**) Schematic diagram of detection of ROS and $O_{2}^{•-}$ probes, respectively. Intracellular ROS production in 4T1 cells treated with (**B**) phosphate buffered saline (PBS) and (**C**) UZNCs + 980-nm laser with DCFH-DA. (**D**) Corresponding M.F.I. of intracellular ROS production in 4T1 cells treated with (I) PBS, (II) ZIF-8 nanoparticles, (III) ZIF-67 nanoparticles, (IV) Zn–Co ZIF nanoparticles, (V) UZNCs and (VI) UZNCs + 980-nm laser. Intracellular $O_{2}^{•-}$ generation of 4T1 cells treated with (**E**) PBS and (**F**) UZNCs + 980-nm laser. DHE was used as the $O_{2}^{•-}$ sensor. (**G**) The corresponding M.F.I. of intracellular $O_{2}^{•-}$ generation of 4T1 cells treated with (I) PBS, (II) ZIF-8 nanoparticles, (III) ZIF-67 nanoparticles, (IV) Zn–Co ZIF nanoparticles, (V) UZNCs and (VI) UZNCs + 980-nm laser. (**H**) CLSM of 4T1 cells after treated with PBS, ZIF-8 nanoparticles, ZIF-67 nanoparticles, Zn–Co ZIF nanoparticles, UZNCs and UZNCs with 980-nm laser irradiation. (**I**) Apoptosis analysis of 4T1 cells treated with PBS, ZIF-8 nanoparticles, ZIF-67 nanoparticles, Zn–Co ZIF nanoparticles, UZNCs and UZNCs + 980-nm laser. ***P < 0.001.

The excellent ^•^OH-generating properties of the above UZNCs in solution motivated us to explore their intercellular ROS-generating effects. To validate this, 4T1 cells treated with the UZNCs nanotherapeutic system were stained with dichlorodihydrofluorescein diacetate (DCFH-DA) (ROS probe). The specific colorimetric mechanism of DCFH-DA for ^•^OH was shown in Fig. 3A. After DCFH-DA was incubated with cells for 1 h, a strong green fluorescence signal could be seen with CLSM. 4T1 cells treated with phosphate buffered saline (PBS) did not show green fluorescence (Fig. 3B), in contrast, the UZNCs + 980 nm group displayed the most intense green fluorescence, signifying the highest ROS production (Fig. 3C). Moreover, the mean fluorescence intensity (MFI) of PBS, ZIF-8 nanoparticles, ZIF-67 nanoparticles, Zn–Co ZIF nanoparticles, UZNCs and UZNCs + 980-nm laser group was also measured (Fig. 3D), and the fluorescence intensity of each group was corresponded to the fluorescence photos (Fig. 3B and C and Supplementary Fig. S10). This further revealed that the bimetallic MOF composed of Zn^2+^ and Co^2+^ as metal nodes can function as a tandem reactor in the complex tumor microenvironment, disrupting the redox equilibrium within tumor cells, thereby stimulating the production of intracellular free radicals, resulting in a substantial increase in intracellular ROS levels. Furthermore, the production of intracellular $O_{2}^{•-}$ was observed by laser confocal microscope, with a DHE probe employed, which could interact with $O_{2}^{•-}$ and emit red fluorescence. There was no red fluorescence observed in the PBS group (Fig. 3E); however, the UZNCs + 980 nm group showed bright red fluorescence (Fig. 3F). Moreover, the fluorescence photos and MFI of each group were also measured (Fig. 3G and Supplementary Fig. S11), showing that Zn^2+^ can promote the production of $O_{2}^{•-}$ in cells. The resulting $O_{2}^{•-}$ could serve as the initiation to disturb the redox balance in tumor cells, thereby carrying out the subsequent Fenton-like reaction to kill tumor cells and offering better efficacy for CDT. To examine the distribution of viable and deceased tumor cells after a 24-h treatment with the nanotherapeutic system, 4T1 cells were marked with the fluorescent dyes and treated with ZIF-67, ZIF-8, Zn–Co ZIF, UZNCs and UZNCs + 980 nm for 24 h. As observed by CLSM, the cell viability was not affected for the control, ZIF-67 and ZIF-8 groups, showing a strong green fluorescence signal. In contrast, after treatment with Zn–Co ZIF, a significant number of 4T1 cells were eradicated, showing a strong red fluorescence signal. More importantly, practically all 4T1 cells were eradicated following treatment with UZNCs and 980-nm laser irradiation (Fig. 3H). The apoptosis rate of the UZNCs + 980 nm group was 99.8%; this was notably higher than the levels observed in the other groups, providing further confirmation that treatment with UZNCs and 980-nm laser irradiation amplified the therapeutic impact of CDT on 4T1 cells (Fig. 3I).

### Evaluation of tumor therapy *in vivo*

**Figure 4.**
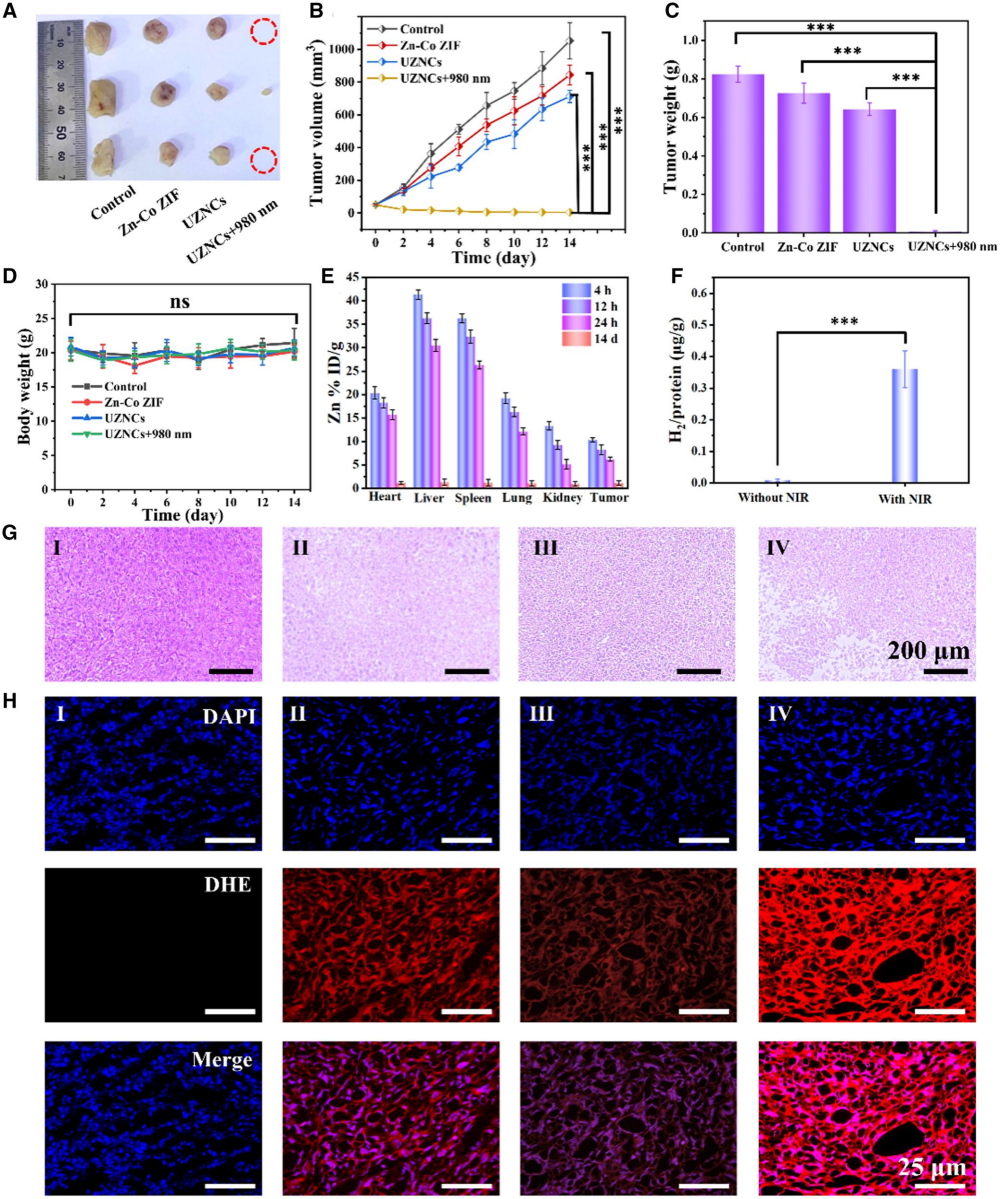
*In vivo* evaluation of antitumor efficacy. (**A**) Photographs of the excised tumors following treatments administered over a 14-day period. (**B**) Alterations in tumor volumes subsequent to diverse treatments. (**C**) The weight of the dissected tumors after different treatments over a 14-day period. (**D**) Changes in the body weight of mice following diverse treatments. (**E**) Biodistribution (liver, tumor, spleen, lung, kidney and heart) of UZNCs. (**F**) The hydrogen levels in tumor mice treated with UZNCs. ***P<0.001. (**G**) DHE staining (red fluorescence) of tumor tissues after treated with (I) phosphate buffered saline (PBS), (II) Zn–Co ZIF nanoparticles, (III) UZNCs and (IV) UZNCs + 980-nm laser. (**H**) H&E staining of tumor tissues after treatment with (I) PBS, (II) Zn–Co ZIF nanoparticles, (III) UZNCs and (IV) UZNCs + 980-nm laser. ***P<0.001.

As UZNCs exhibited excellent subcellular H_2_ production and CDT efficacy *in vitro*, the impact of combined CDT and gas therapy was examined in mice bearing 4T1 tumors (Fig. 4A–C). The tandem reactor composed of Zn^2+^ and Co^2+^ ions can break the original redox balance in tumor cells, resulting in a sharp increase in intracellular ROS content, thereby achieving good CDT. Results showed that the tumor growth was slightly inhibited by Zn–Co ZIF nanoparticles, which could be attributed to the establishment of a tandem reactor involving Zn^2+^ and Co^2+^ released from Zn–Co ZIF nanoparticles at the tumor site. The results of the UZNCs group without light treatment did not exhibit significant differences from the Zn–Co ZIF group. This suggests that UCNP cores had no discernible anti-cancer effect in the absence of an external light source. In comparison, the UZNCs + 980 nm group significantly inhibited tumor growth in mice, attributing to the combined treatment of CDT/H_2_ induced by UZNCs: H_2_ produced after UCNPs uptake of NIR caused damage to cellular mitochondria, inhibiting their normal ATP synthesis activities, thus making cells more vulnerable; simultaneously, the presence of Zn^2+^ and Co^2+^ in the tandem reactor could disrupt the redox balance within the tumor cells, leading to the generation of a substantial amount of ROS, thereby achieving a more effective anti-tumor effect. In addition, no significant changes in body weight were observed for the four groups of mice, control, Zn–Co ZIF, UZNCs and UZNCs + 980 nm, within 14 days of observation (Fig. 4D). To evaluate the specific retention ability of UZNCs at tumor sites and its residual rate in various organs, we treated 4T1 tumor mice with UZNCs and employed inductively coupled plasma-mass spectrometry (ICP-MS) to test the concentration of metal ion at the tumor sites. Figure 4E shows the ion concentrations enriched in different organs over the time. ICP-MS results from tumor homogenate showed that the ion concentration gradually increased after intravenous injection, which was related to the circulation of UZNCs in mice. Nevertheless, after 4 h post-injection, the ion concentration reached the maximum intensity and then gradually weakened, and UZNCs hardly remained in the tumor and major organs after 14 days of intravenous injection, demonstrating its high excretion rate. Next, we investigated the H_2_ production of UZNCs at the tumor site. The amount of H_2_ at the tumor site was determined by gas chromatography after fragmentation and ablation. Results indicate that after 980-nm laser illumination, UZNCs generated a large amount of hydrogen for the favorable hydrogen therapeutic efficacy (Fig. 4F). H&E experiments showed that the UZNCs could promote cancer cell apoptosis through CDT/H_2_ combined therapy (Fig. 4G). We also studied the $O_{2}^{•-}$ generated at tumor sites in different groups and found that compared with control, the other three groups had strong red fluorescence, showing the high ROS levels due to that Zn^2+^ could indeed generate $O_{2}^{•-}$ at the tumor site, paving the way for the subsequent disturbance of redox balance in tumor cells to generate ROS (Fig. 4H and Supplementary Fig. S12). H&E staining in each group showed that no lesions occurred in each tissue, indicating that normal tissues were not damaged during the treatment process, proving their good biocompatibility (Supplementary Fig. S13). Together, these results demonstrated that UZNCs exhibited excellent therapeutic efficacy with high biocompatibility and biosafety. *In vivo* experiments results showed that NIR photocatalysis can cause an increase in the level of ROS in tumor cells, thereby damaging tumor cell DNA and inducing tumor cell apoptosis; simultaneously, the H_2_ generated by NIR photocatalysis could reduce tumor cell resistance against ROS, preventing DNA repair, accelerating tumor cell apoptosis and elevating the overall therapeutic efficacy.

## Conclusion

To summarize, we have fabricated a nanozyme-based bimetallic MOFs tandem reactor, UZNCs, to elevate intracellular H_2_O_2_ levels, improving cell susceptibility and thereby enhancing CDT efficacy. UCNPs loaded in UZNCs served as the nanotransducers converting NIR to blue/UV light for the H_2_ generation. The generated H_2_ molecule could starve cancer cells, damage DNA, induce tumor cell apoptosis and alleviate the cancer cells’ susceptibility to CDT. With low toxicity and high therapeutic efficacy, we believe that the proposed UZNCs-mediated CDT and H_2_ therapy could serve as a new avenue for achieving highly efficient and low-toxic cancer therapy.

## Supplementary Material

rbad097_Supplementary_Data
