# Supplementary material for: NIR-switchable local hydrogen generation by tandem bimetallic MOFs nanocomposites for enhanced chemodynamic therapy
Source: Regen Biomater. 2023 Oct 31;11:rbad097. doi: 10.1093/rb/rbad097 (PMC10761206; doi:10.1093/rb/rbad097)
Supplement: rbad097_Supplementary_Data [file rbad097_supplementary_data.docx]

Supplementary data

**NIR-switchable Local Hydrogen Generation by Tandem Bimetallic MOFs Nanocomposites for Enhanced Chemodynamic Therapy**

**Jun Zhong^1,2^, Xiang Zheng^1,2*^, Yuan Wen^1,2^, Yuewei Li^1,2^, Jianting Zhang^1,2^, Ranjith Kumar Kankala^1,2^, Shibin Wang^1,2^ and Aizheng Chen^1,2,3^***

^1^Institute of Biomaterials and Tissue Engineering, Huaqiao University, Xiamen 361021, P. R. China.

^2^Fujian Provincial Key Laboratory of Biochemical Technology, Huaqiao University, Xiamen 361021, P. R. China.

^3^Fujian Provincial Key Laboratory of Biomass Low-Carbon Conversion, Huaqiao University, Xiamen 361021, P.R. China.

*Corresponding author

E-mail address: [zhengxiang@hqu.edu.cn](mailto:zhengxiang@hqu.edu.cn) [azchen@hqu.edu.cn](mailto:azchen@hqu.edu.cn)


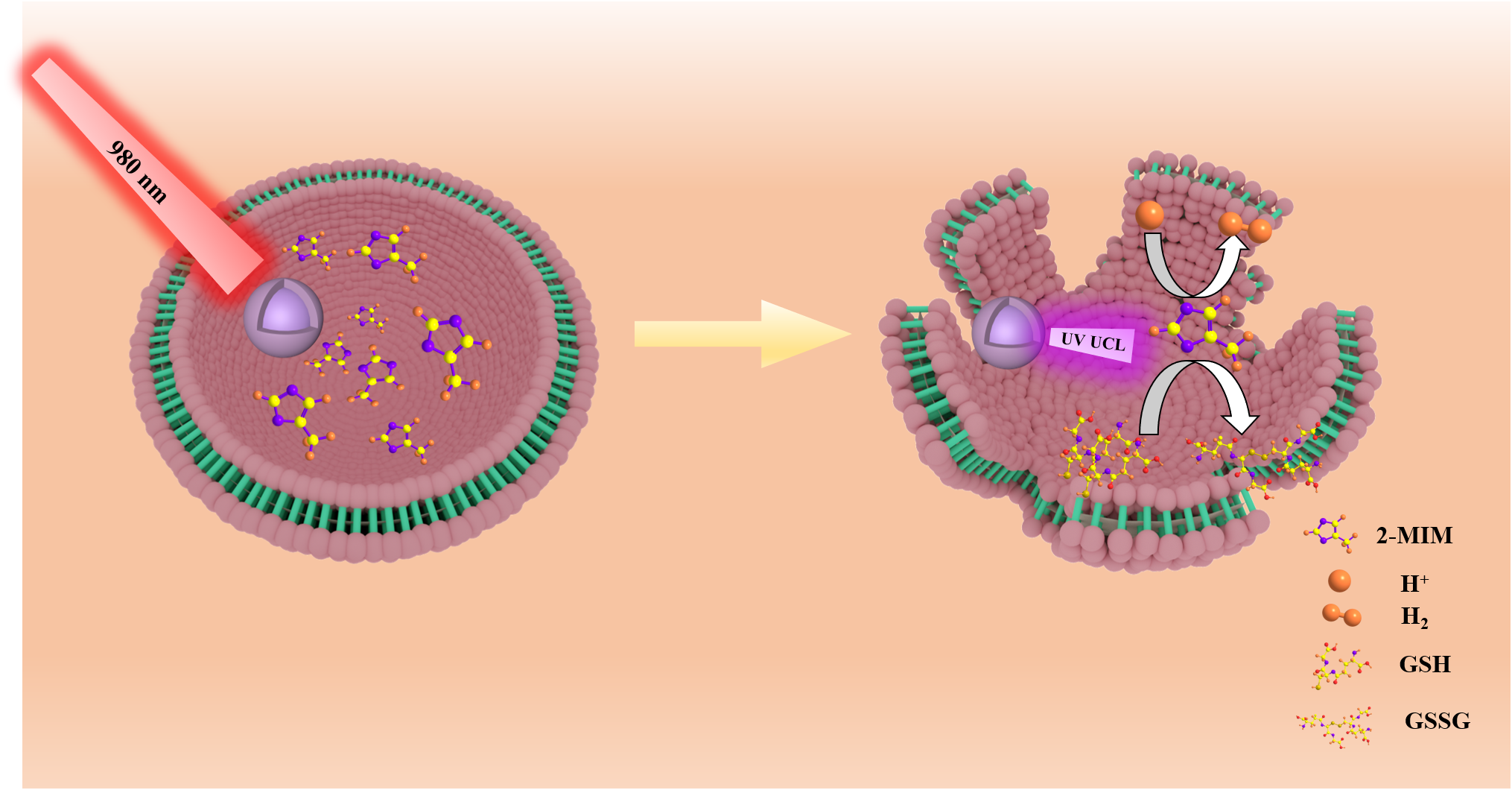


**Figure S1.** Schematic illustration of UCNPs-mediated hydrogen generation to facilitate lysosomal escape of nanoparticles.


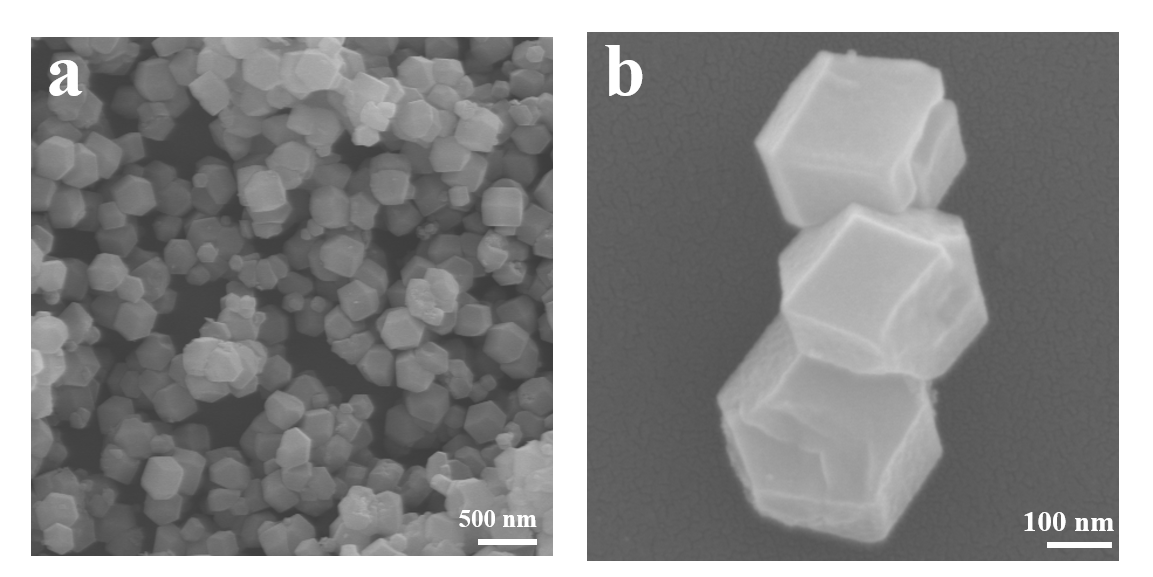


**Figure S2.** SEM images of Zn-Co ZIF with (a) low magnification and (b) high magnification.


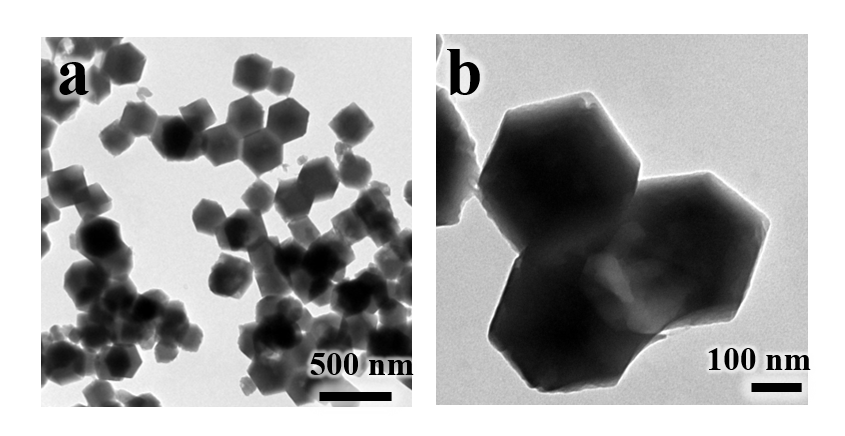


**Figure S3.** TEM images of Zn-Co ZIF with (a) low magnification and (b) high magnification.


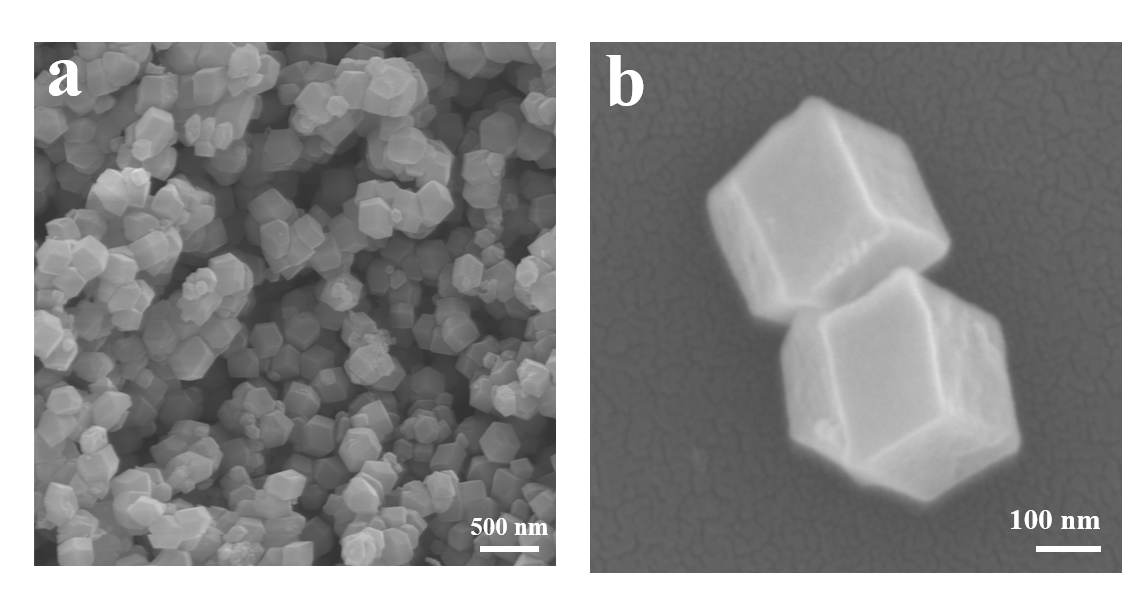


**Figure S4.** SEM images of UZNCs with (a) low magnification and (b) high magnification.


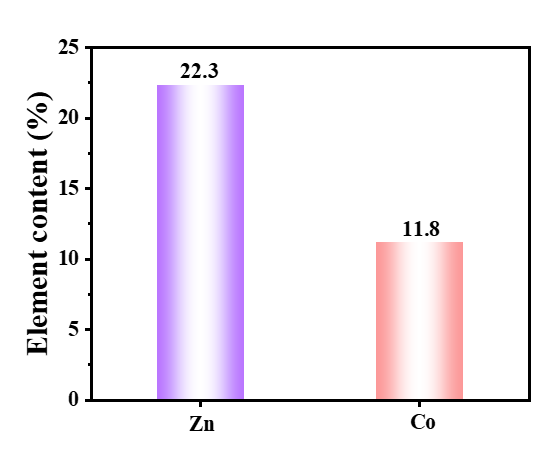


**Figure S5.** The element contents of Zn and Co in Zn-Co ZIF nanoparticles test by ICP-MS.





**Figure S6.** The hydrodynamic diameters of Zn-Co ZIF and UZNC.





**Figure S7.** N_2_ adsorption–desorption isotherms of Zn-Co ZIF and UZNC.


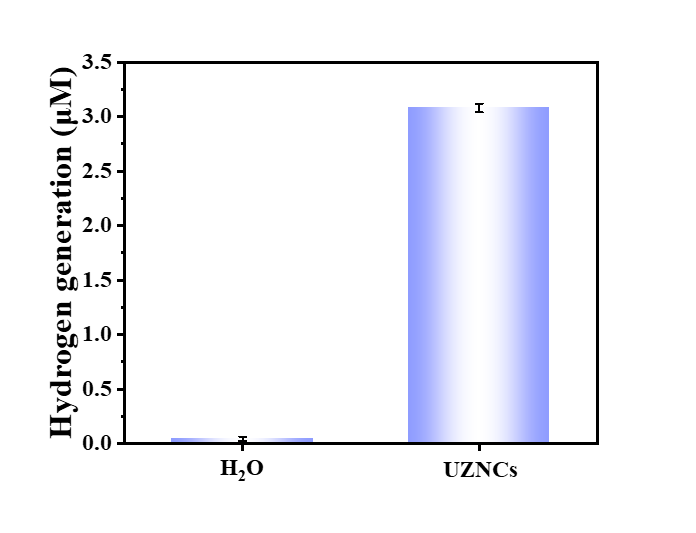


**Figure S8.** Hydrogen production performance of UZNCs in GSH (10 μM) solution.


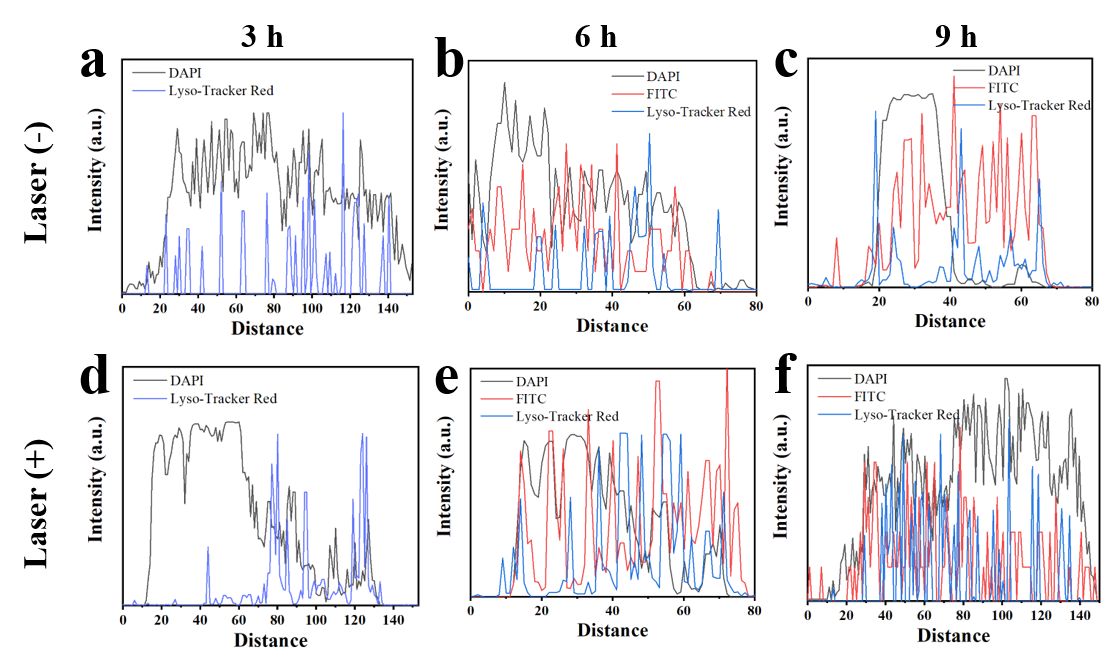


**Figure S9.** Linear section fluorescence intensity profile of LysoTracker Red (blue line), UZNCs -FITC (red line), and DAPI (black line). UZNCs -FITC was incubated with 4T1 cells without NIR light for (a) 3 h, (b) 6 h, and (c) 9 h. (d-f) UZNCs -FITC was incubated with 4T1 cells after NIR light for (a) 3 h, (b) 6 h, and (c) 9 h.


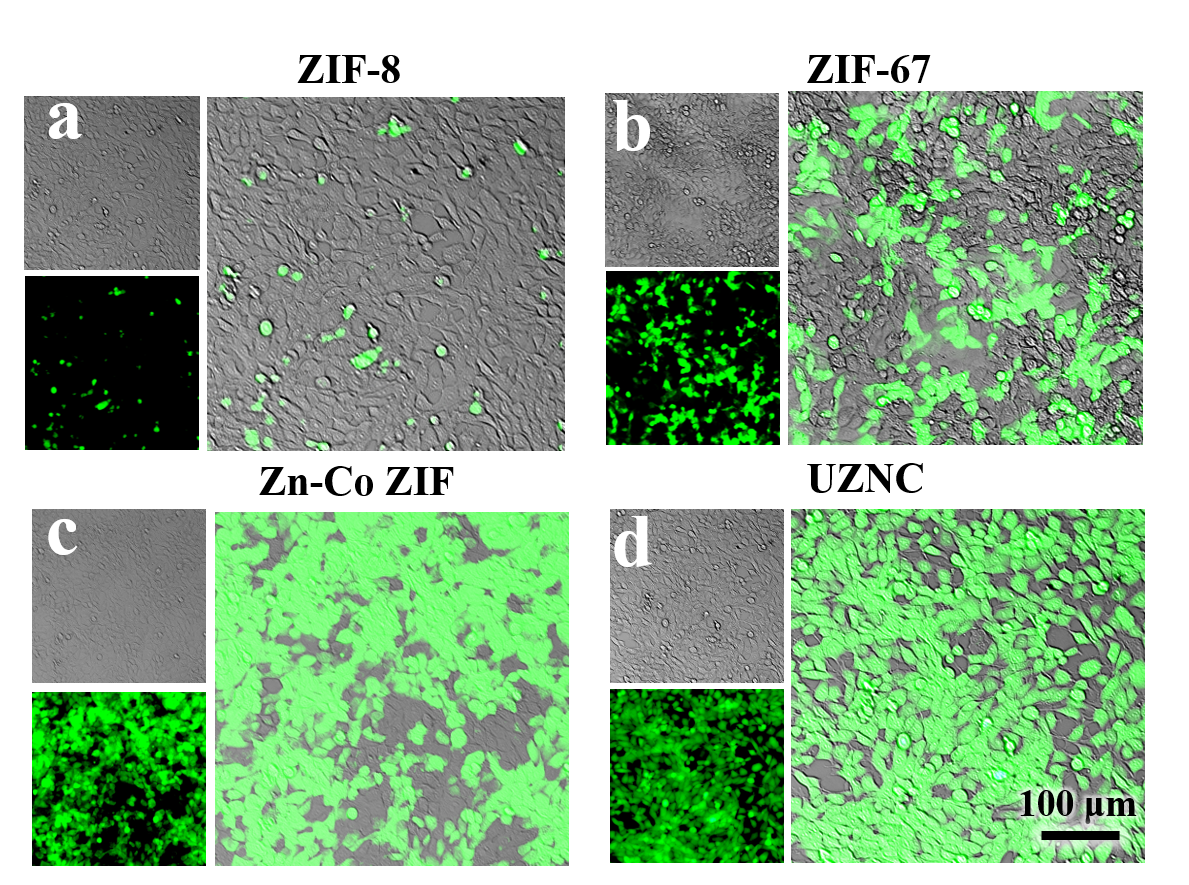


**Figure S10.** Intracellular ROS generation of 4T1 cells treated with (a) ZIF-8 nanoparticles, (b) ZIF-67 nanoparticles, (c) Zn-Co ZIF nanoparticles, and (d) UZNCs. DCFH-DA was used as the ROS sensor.


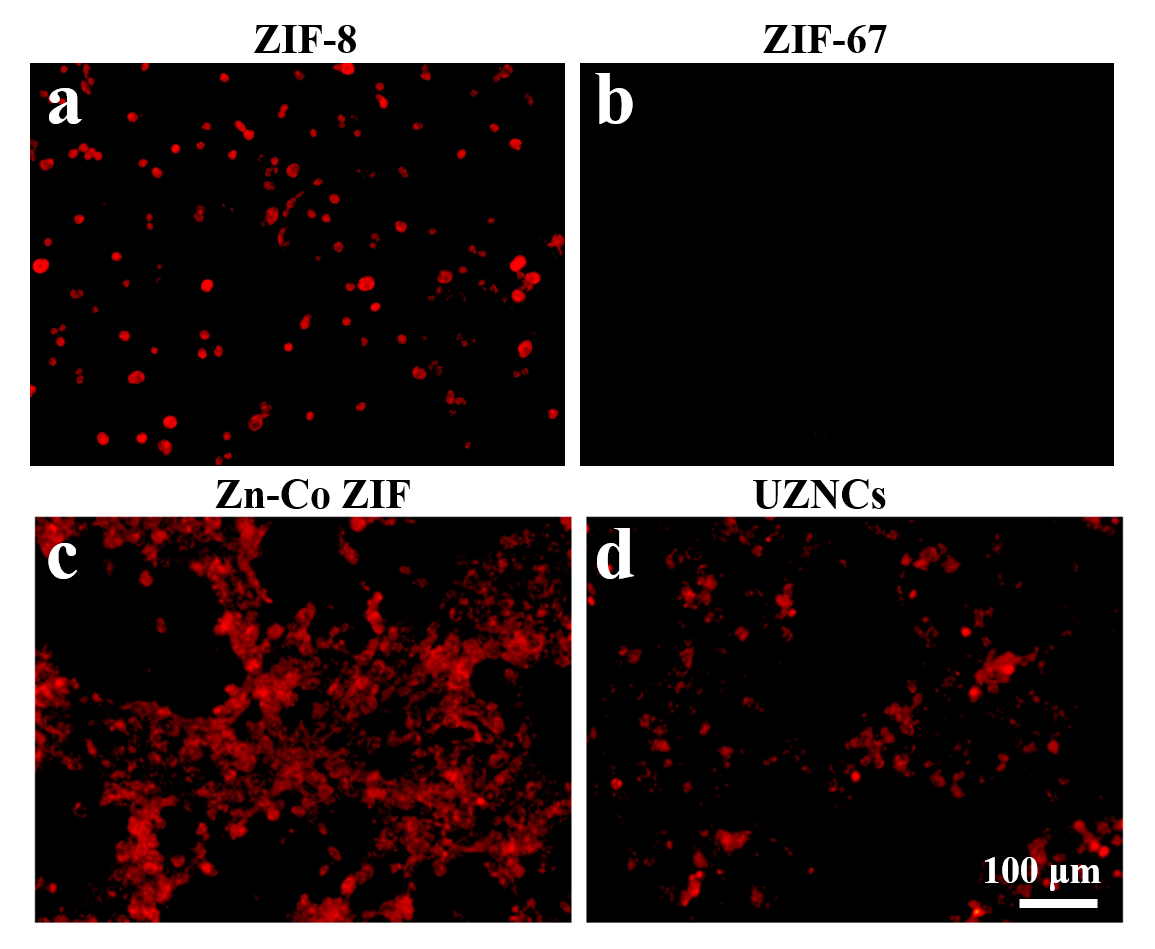


**Figure S11.** Intracellular O_2_^•-^ generation of 4T1 cells treated with (a) ZIF-8 nanoparticles, (b) ZIF-67 nanoparticles, (c) Zn-Co ZIF nanoparticles, and (d) UZNCs. DHE was used as the O_2_^•-^ sensor.


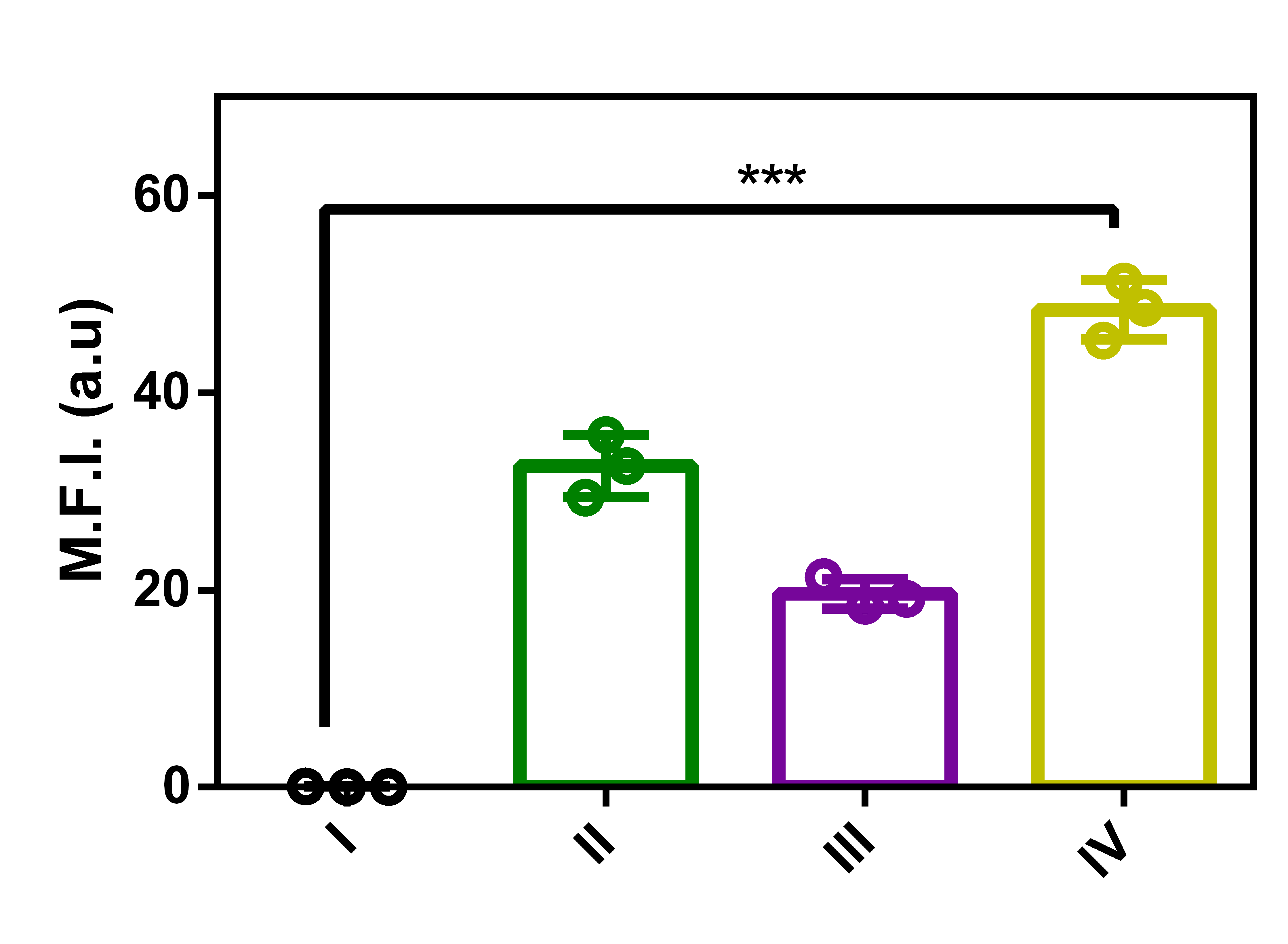


**Figure S12.** The corresponding MFI of tumor tissues’ DHE staining after treated with (I) PBS, (II) Zn-Co ZIF nanoparticles, (III) UZNCs and (IV) UZNCs + 980 nm laser. Data were expressed as mean ± standard deviation (n = 3 per group). ***p < 0.001


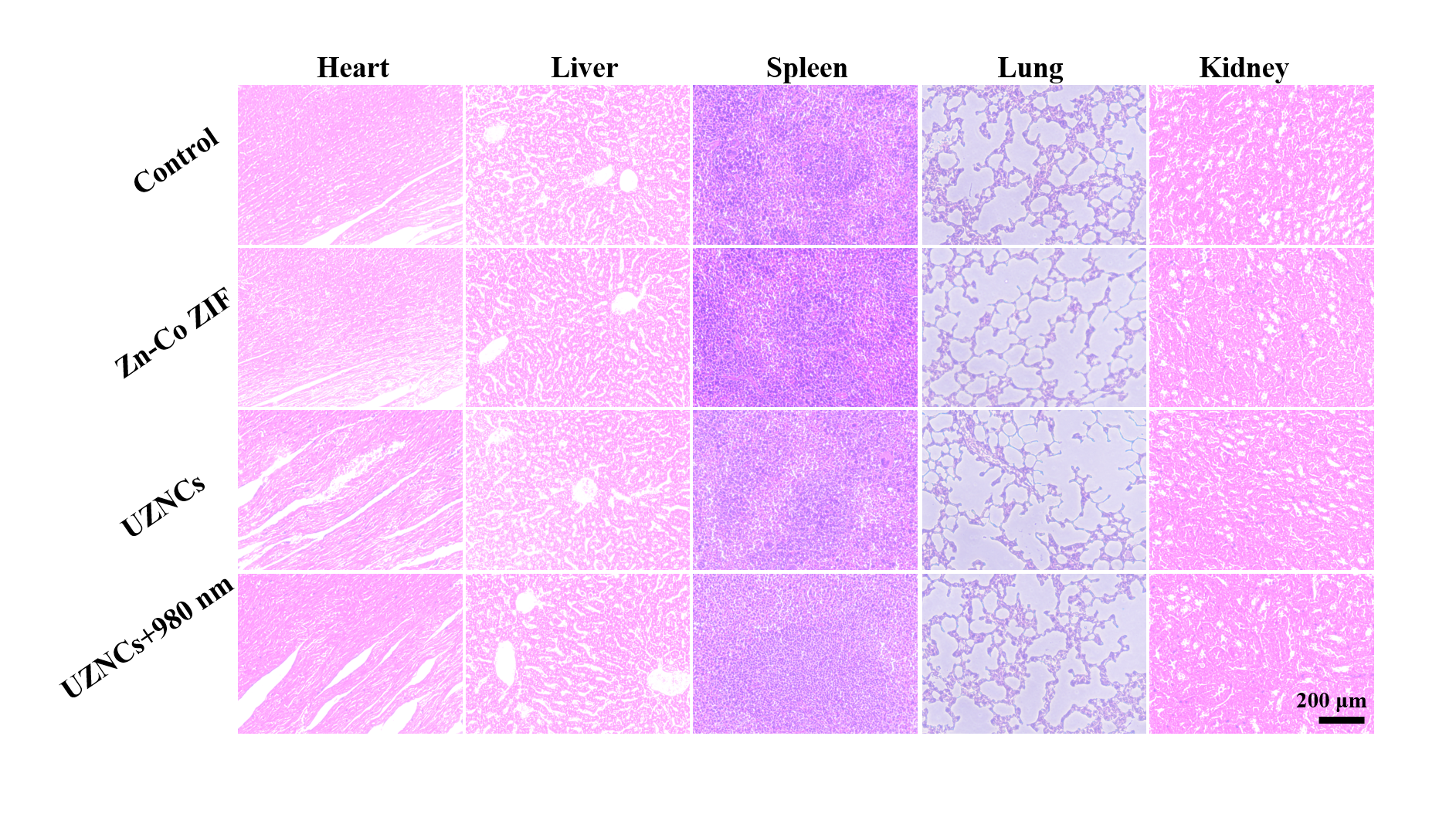


**Figure S13.** H&E staining of major organs.
